# Case report: Rapid improvement of crossed cerebellar diaschisis after intravascular laser irradiation of blood in a case of stroke

**DOI:** 10.1097/MD.0000000000005646

**Published:** 2017-01-13

**Authors:** Wan-Hua Yang, Shiou-Ping Lin, Shin-Tsu Chang

**Affiliations:** Department of Physical Medicine and Rehabilitation, Taichung Veterans General Hospital, Taichung, Taiwan.

**Keywords:** case report, crossed cerebellar diaschisis, intravascular laser irradiation, stroke

## Abstract

**Rationale:**

Crossed cerebellar diaschisis (CCD) is a poor prognostic factor after stroke because without immediate cerebral reperfusion no further improvements in the patient's condition can be achieved. We investigated the clinical effects of intravascular laser irradiation therapy (ILIB) on CCD and evaluated the therapeutic effect in the sub-acute post-stroke stage.

**Patient concerns:**

The 77-year-old male with cerebral infarction in the territory of the right anterior cerebral artery only underwent conservative treatment including hydration and aspirin in the acute post-stroke stage.

**Diagnosis:**

He was diagnosed as stroke based on the clinical presentations and imaging findings.

**Intervention:**

Once the patient was in stable condition, he underwent a daily hour-long ILIB (He-Ne laser) for ten consecutive days during the sub-acute post-stroke stage.

**Outcomes:**

We used single-photon emission computed tomography (SPECT) before and after intravascular laser irradiation to detect changes in cerebral and cerebellar perfusion. Then, we compared the two images. CCD was detected using the first SPECT. After intervention by ILIB, the second SPECT showed greater perfusion in the affected cerebellar hemisphere.

**Lessons:**

We found that ILIB helped eliminate CCD, which was previously shown to be an untreatable condition using any intervention during the sub-acute post-stroke stage. Stroke patients could therefore greatly benefit from ILIB.

## 1 Introduction

Crossed cerebellar diaschisis (CCD) is the decrease of blood flow and metabolism of a region in the cerebellar hemisphere contralateral to the supratentorial stroke.^[1]^ CCD leads to functional disconnection of the contralateral hemisphere from the cerebral cortex and cannot be eradicated. Many studies indicated that CCD could be a poor prognostic factor or an outcome predictor after stroke events. Patients with CCD also have worse functional scales including Barthel index.^[2]^ Thus, it is necessary to develop a new intervention to eliminate CCD, with a view to improving outcomes in patients with stroke.

Low-level laser therapy (LLLT) is widely used in clinical treatment for many pathological processes such as pain control, insomnia, metabolic diseases, spinal cord injury, traumatic brain injury, and stroke by promotion of mitochondrial functions.^[3]^ Intravascular laser irradiation of blood (ILIB) is also a low-power therapeutic tool that is applied in many clinical events to facilitate circulation. Therefore, we postulated that ILIB would improve cerebral circulation thereby eliminating CCD. In this report, we present a case with unilateral cerebral infarction accompanied by CCD, who received ILIB treatment. Postprocedural imaging study revealed that the procedure was effective in eliminating CCD.

## 2 Case presentation

A 77-year-old male, with progressive weakness of left limbs, suffered from falling, choking, and slow response. He was sent to our emergency room without thrombolytic therapy because the critical first 3 h had passed. His muscle power was determined as grade 5 in both upper and lower right extremities, grade 1 in the proximal upper left extremity, and grade 2 in the lower left extremity. Emergent computed tomography of the brain showed no brain hemorrhage. After admission, his muscle power reduced to grade 1 in the lower left extremities. Scores on the Glasgow coma scale were as follows: eye opening response, 4; verbal response, 2; and, motor response, 6. The second computed tomography of the brain, which was conducted on October 9, 2014, revealed low density over the right anterior cerebral artery, compatible with recent infarction symptoms.

**Figure 1:**
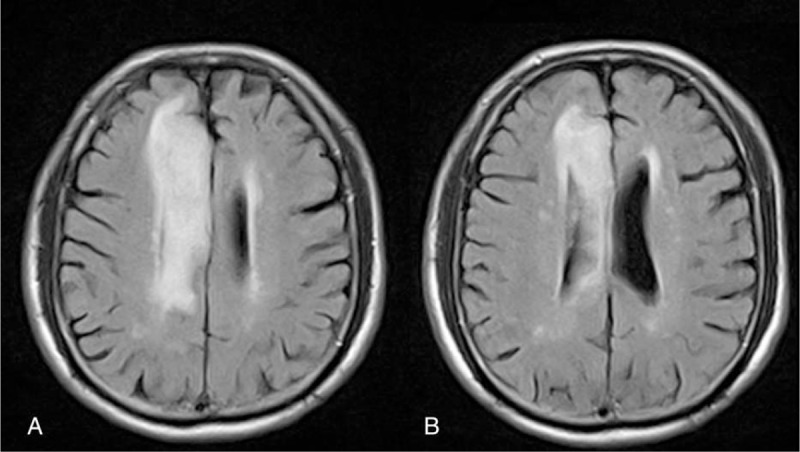
Brain MRI. (A and B) Serial FLAIR MRI shows the high signal intensity in the territory of the right anterior cerebral artery, which indicates recent cerebral infarction. MRI = magnetic resonance imaging.

**Figure 2:**
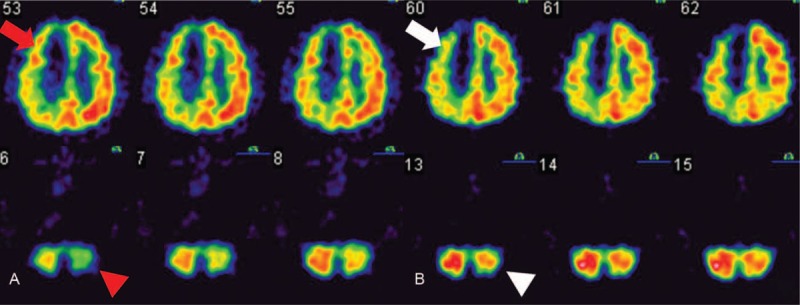
Regional perfusion SPECT before and after intravascular laser irradiation. The 2 SPECT images show the regional perfusion. The white to red area indicated better perfusion than the blue and green area. (A) This regional perfusion SPECT (conducted on poststroke day 14) shows that the infarction region (indicated by red arrow) covers the territory of the anterior cerebral artery on the right hemisphere and decreased perfusion on the left cerebellar hemisphere (indicated by red arrow head). (B) The second regional perfusion SPECT (conducted on poststroke day 63) still showed hypoperfusion in the right cerebral hemisphere (indicated by white arrow), but more blood flow in the left cerebellar hemisphere (indicated by white arrow head). SPECT = single-photon emission computed tomography.

The results of the brain magnetic resonance imaging study disclosed an infarction in the region of the right anterior cerebral artery (Fig. 1). Contralateral hemiplegia, grasp reflex, akinetic mutism, and urinary retention were noted after the stroke. In addition, a comprehensive stroke survey was completed which revealed type 2 diabetes mellitus and dyslipidemia. Meanwhile, aspirin 25 mg, dipyridamole 200 mg (aggrenox, 225 mg, Boehringer Ingelheim, Taipei City), metformin (glucophage, 500 mg, Merck, Taipei City, Taiwan [ROC]), and atorvastatin (Lipitor, 10 mg, pfrizer, New Taipei City, Taiwan [ROC]) were prescribed for stroke, type 2 diabetes mellitus, and dyslipidemia, respectively. After recovery, once his condition was deemed to be relatively stable, he received rehabilitation in our ward. The initial Barthel index scores were as follows: eating (5), grooming (0), dressing (5), toileting (0), bathing (0), bladder (5), bowel (5), stairs (0), transfer (0), and ambulation (0). The brain regional perfusion was imaged by single-photon emission computed tomography (SPECT) (Fig. 2A) 2 weeks after the stroke and disclosed CCD on the left side. The patient's family was eager to try novel approaches to promote neurologic recovery and thus we opted to use ILIB therapeutically.

### 2.1 Subject

**Figure 3:**
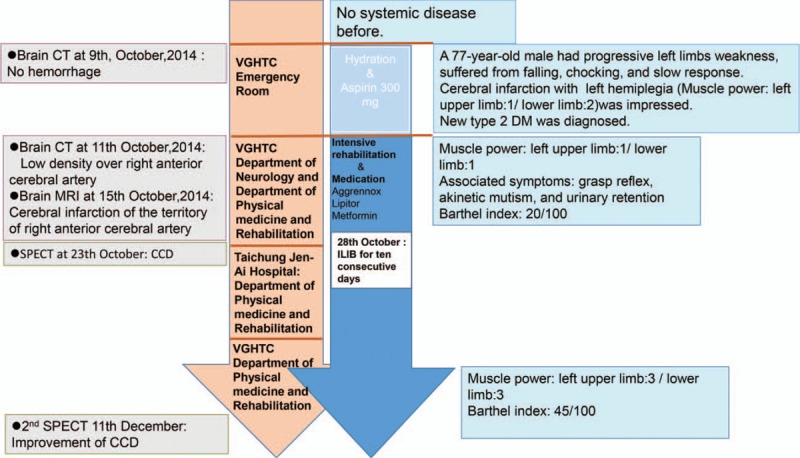
Timeline. The timeline containing the interventions, image reports, and general conditions provided the time course of this patient we presented.

This patient who was newly diagnosed with unilateral stroke, type 2 diabetes mellitus, and dyslipidemia has taken medications to control metabolism disease and prevent another stroke attack since acute poststroke stage. There were no life threatened complications such as respiratory failure but left hemiplegia. Intensive rehabilitation was started in the acute poststroke stage during admission. At our ward, the first SPECT was performed to observe the cerebral perfusion and disclosed CCD on the left side. We summarized the patient's condition in the timeline (Fig. 3).

### 2.2 Treatment plans

ILIB is a self-paid, alternative treatment for metabolic disease, insomnia, and pain control in Taiwan. Permission to use ILIB was provided by the patient's son. The patient and his son understood and accepted the therapy and possible side effects. ILIB is operated as follows: our medical team finds an adequate vein (bilateral median antebrachial vein, cephalic vein, accessory cephalic vein by turns), which is suitable for intravascular insertion firstly. After tightening the tourniquet above the site of the selected vein and sterile procedure is done on the insertion site, a catheter is inserted into the vein by a needle, which is subsequently removed while a cannula is remained in the place. Then, a fiber is inserted into the cannula that provides a channel for laser passing through. We adopted the helium–neon (HeNe) laser (YJ-ILIB-5, Bio-ILIB Human Energy Ltd, Taiwan) in this study. The patient assigned to ILIB was treated with 632.8 nm wavelength HeNe, power output 3.5 to 4.0 w/cm^2^, power intensity 1.79 to 2.04 w/cm^2^, energy 12.6 to 14.4 J, energy density 6428.57 J/cm^2^, and irradiation time 3600 s/ session. We titrated up the power output gradually according to the patient's clinical response instead of the constant power output. The patient received a complete course of ILIB lasting an hour for 10 consecutive days.

We conducted 2 images of SPECT before and after ILIB to detect the change of cerebral perfusion. Also, functional ability was determined by Barthel index, and Brunnstrom stage and muscle power were evaluate before and after ILIB.

The second SPECT study was performed 63 days after the stroke event, and the regional perfusion showed that CCD had greatly improved (Fig. 2B). Comparing the 2 regional perfusion SPECT data, we found that the blood perfusion in the right cerebellar hemisphere showed greater resemblance to the left cerebellar hemisphere in the second SPECT after ILIB. The SPECT data also revealed that the CCD was decreased in the patient after ILIB. In addition, the patient appeared to be more energetic and muscle power was improved to grade 3 in the left extremities. His Brunnstrom stage was determined to be III in the proximal upper left extremity/III of the left hand/III in the lower left extremity. He attained functional recovery, which was quantified by the Barthel index: eating (10), grooming (0), dressing (5), toileting (5), bathing (0), bladder (10), bowel (10), stairs (0), transfer (5), and ambulation (10). Taken together, these results of the present case indicated that ILIB may be a useful tool for improving outcomes in patients with CCD following a stroke.

## 3 Discussion

CCD is a regional hypoperfusion in the cerebellar hemisphere, contralateral to the supratentorial stroke region that can be easily detected by SPECT.^[1]^ The widely accepted mechanism of CCD is the disturbance of the cortico-ponto-cerebellar pathway by a depressed metabolism and decreased blood flow following the occurrence of a cerebral lesion, such as cerebral infarction and other injuries.^[4]^ In addition, CCD is considered to be a broadly and persistent phenomenon in patients after stroke attack.

The occurrence of CCD is a source of considerable frustration for both patients and their care providers. A previous study indicated that CCD disappeared when patients received nutritional reperfusion by thrombolytic agents that were ordered within the golden hour.^[5]^ However, the patient in the present case did not receive thrombolytic agents as the onset of symptoms after the stroke attack was gradual and therefore treatment was not sought immediately following the stroke. The first SPECT showed a hypoperfusion in the territory of the right anterior cerebral artery combined with CCD in the left cerebellum. Thus, we have reviewed several studies, which disclosed LLLT could promote the circulation of central nervous systems even facilitate new neuronal cell formation.^[6,7]^ Thus, we hypothesize that LLLT could promote the cerebral circulation in the stroke patient and thereby eradicate CCD. ILIB, a kind of LLLT, was available at our department. Thus, ILIB was applied to this stroke we presented in this case. Remarkably, one-and-a-half months of intravascular laser therapy using HeNe laser improved CCD in the subacute poststroke stage, according to the results of the postirradiation SPECT imaging study. Furthermore, the patient showed improved emotional output and better activity daily of living function compared with that of the initial evaluation by Barthel index. To the best of our knowledge, this is the first report to show that CCD in a stroke patient in the subacute stage was improved after intravascular laser therapy.

Several studies have investigated the mechanism of neurological recovery after intravascular laser therapy in order to understand how it promotes cerebral blood flow. In 1 investigation, less functional deficiency was found in patients who received transcranial infrared laser therapy with a laser wavelength of 808 nm, which was initiated within 24 h of stroke onset, compared with controls.^[6]^ A recent study also found that new neuronal cells were generated in the subventricular zone of the infarcted hemisphere in stroke rats which received transcranial LLLT, and fewer neurological deficits were also noted.^[7]^ The effectiveness of neurologic recovery following intervention using LLLT was demonstrated in these studies. The most widely accepted mechanism of LLLT involves well-understood intracellular processes with Cytochrome c, a photoacceptor, which can be stimulated by LLLT, promotes adenosine triphosphate (ATP) production to enhance the efficacy of the respiratory chain in the mitochondria.^[8]^ That indicated LLLT may be an effective way via increasing cellular metabolism and decreasing the generation of free radicals in injured tissues. Furthermore, according to a study on the use of ILIB to treat patients with spinal cord injury, ILIB was shown to alter ATP synthesis in white blood cells and reduce low-density lipoprotein.^[9]^

The limitation of the presented case was difficult to clarify whether the effects of neuronal and functional recovery were mediated by intensive rehabilitation, ILIB or natural course. In fact, it was very difficult to validate the level of benefits in a stroke patient from intensive rehabilitation due to individual severities, comorbidities, age, and motivation. Intensive rehabilitation is a regular treatment that could improve body functions of patients with stroke. Therefore, in the presented case, to optimize the most benefits for the patient, we still arranged intensive rehabilitation and combined ILIB for him during admission. CCD could not be eradicated without nutritional cerebral reperfusion in the very acute poststroke stage once. To date, there were no effective treatments to improve and/or eradicate CCD in the subacute poststroke stage before our case report. There were no available reports suggested that intensive rehabilitation could improve cerebral perfusion in the subacute poststroke stage. In addition, some studies indicated that LLLT promoted cerebral perfusion and formation of neuron cells.^[6,7]^ Thus, we postulated that stroke patients with CCD should get benefits from ILIB. Besides, there was no appropriate and objective parameter to validate the functional recovery. To measure the changes of cerebral and cerebellar perfusion after ILIB, SPECT could be a desirable tool to provide the objective data with imaging the cerebral perfusion. Now, we found clear evidence of greatly improved cerebellar perfusion on the affected side using SPECT. CCD, which was once could not be improved in the subacute poststroke stage, was decreased after ILIB. Moreover, in this study, the patient exhibited far greater functional recovery after ILIB than that typically seen in poststroke patients in our experience. This prior case study presented combination of ILIB and intensive rehabilitation exerted obviously promotion of cerebral perfusion in the patient.

We had reviewed many studies about treatment and influence of CCD. CCD was a poor prognostic factor after stroke.^[2]^ Decreased of CCD would benefit the patients. Thrombolytic agents could eradicate CCD when patients received nutritional perfusion only in the very acute poststroke stage.^[5]^ But there was no effective treatment as acute poststroke stage pass. The stroke patients who could not be diagnosed quickly would miss their opportunities to receive thrombolytic agent easily. To our knowledge, there were possible complications such as parenchymal hemorrhage after application of thrombolytic agents. Patients with stroke were sent to intensive care unit for further observation and care after thrombolytic agents. However, our case report revealed that CCD could be eradicated in the subacute poststroke stage by ILIB. Current studies had reported if ILIB was applied adequately, ILIB could be more safety treatment than thrombolytic agents. The patients will not be sent to intensive care unit for observation. After that intensive rehabilitation program could be continued. Taken together of these advantages, we hope that ILIB could be an alternative treatment for stroke patients with CCD in the subacute poststroke and make more benefits for them.

## 4 Conclusion

Currently, there is no effective intervention at the subacute poststroke stage capable of improving the outcomes of stroke patients. In this case report, we found that ILIB could be administered at the subacute poststroke stage in an elderly patient, resulting in considerable improvements in CCD and good functional recovery. We postulate that this intervention may enable even better neurologic recovery if it can be applied earlier, for instance, in the acute stage.
